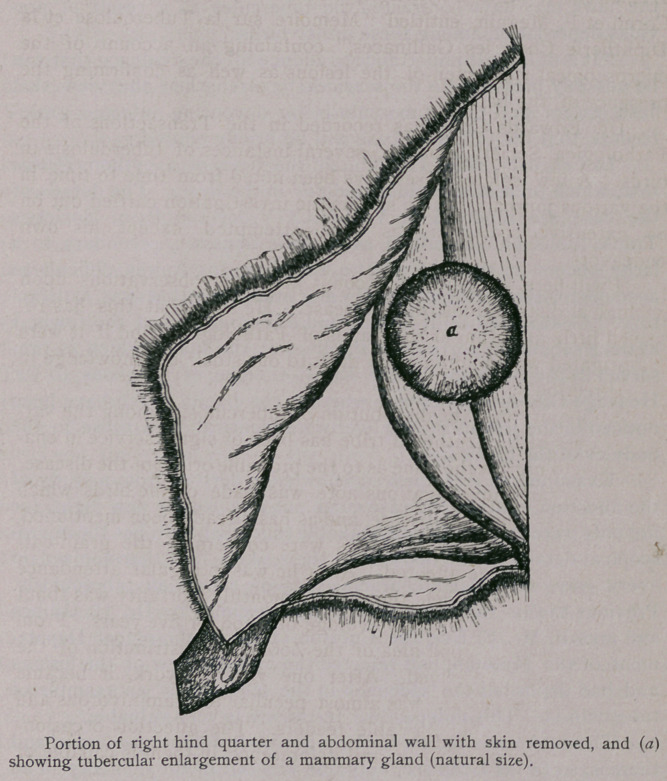# Review of the Avian Tuberculosis

**Published:** 1892-07

**Authors:** S. E. Weber


### REVIEW OF THE AVIAN TUBERCULOSIS.

By S. E. Weber, V. S.

It will not be out of place to give, in connection with my article in the June number of the Journal, a few lines from the admirable article upon Avian Tuberculosis, from the pen of John Bland Sutton, F. R. C. S. (Sir Erasmus Wilson, Lecturer on Pathology, Royal College of Surgeons, Eng.)* in which he describes the pathological lesions, such as in the spleen, bowels, liver, etc., which are identical with those as I found them in rats. He says : "It is a fact well recognized that the inhabitants of certain regions of the earth suffer from diseases which are rare, or even unknown in other parts, some affections are known to be endemic within even a limited area, whilst others are more or less peculiar to certain races of mankind independent of locality. Thus disease has an ethnological as well as geographical distribution."

There is, in addition, a zoölogical distribution of disease, that is to say, every great group of animals suffers, to a large extent, from some affections more than others ; these may not inaptly be termed the "scourges" of the group. Further, if a disease is a scourge to two groups of animals, the lesions in both cases differ in their manifestations in important particulars. This may be partly due to structural peculiarities as well as to differences in the environment, whilst the specific irritant remains the same. The term "irritant" is employed to indicate any substance capable of initiating the inflammatory process. As we shall see later, the scourges are the result of the influence of specific irritants which have a predilection for certain groups of animals, and, in some cases, for particular orders of a group.

There is little doubt that the majority, if not all these scourges, are due to the presence of living matter, either animal or vegetable, which thrives on the tissues of living forms. This is merely an extension of parasitism, for as we know Tænia echinococcus is common to wolves and dogs, Tænia medio-cannelata to sheep and oxen, Cœnurus cerebralis flourishes in the brain of sheep, and some species

* This Journal, Vol. VII., p. 329.

of Acari are only found on the ears of bats; these are merely familiar instances out of a long list that could be adduced.

To return to the affections or scourg, es he further says that syphilis, typhoid fever and tuberculosis are scourges of the human race. The first has never been found except in man. The second, typhoid fever,* has been described in monkeys and a few animals other than man. Tuberculosis though widely distributed, nevertheless devastates the human almost as extensively as any other species. Among the Equidæ, glanders and anthrax are notorious affections, whilst asses are infested to an alarming extent by the worm strongylus armatus. Walley speaks of Eczema epizootica, Pearlsucht, Pleuro-pneumonia, and Rinderpest as the four "bovine scourges." In his article he gives a most elaborate account of a remarkable, wide-spread, and fatal disease affecting more particularly grain-eating birds, and is familiarly known as Avian Tuberculosis. The affection he describes as the scourge of this section of the feathered tribe. His (Bland Sutton's) researches into the nature of this disease commenced in the spring of 1879. "A farmer having lost a large number of fowls in a very short time, requested him to investigate the cause of death, at the same time gave him permission to use the remaining birds in any manner likely to facilitate the inquiry. In 1881 a second outbreak occurred which affected chiefly the young birds. In this, as in the preceding epidemic, only grain-eating birds were affected, ducks and geese escaping entirely. In the meantime fowls from other poultry yards had been furnished him, and it soon became evident that the disease was widely spread in England, for specimens were received from Leeds and Middlesborough in Yorkshire, from Kent, very many places in Middlesex, and from Didcot and Hagbourne in Berkshire. The occurrence of Tuberculosis in these places may be regarded as showing that it is probably met with in most parts of England."

In 1881 he began attending the Zoölogical Gardens of London, and soon found the disease to be very common. For two years observations were made to determine the anatomical and zoölogical distribution of the disease, as determined by the analysis of the cause of death in one thousand birds of various species. In the meantime, Dr. Heneage Gibbes had joined him in his work and for the purpose of determining the relation of the bacilli to the lesions. In the following November (1883) they both made a joint communication to the Pathological Society of London. Since that date many remarkable cases have come under his observation.

*Path. Soc. Trans., Vol. XXXVI, p. 527, and this Journal, Vol. VII, p. 218.

(*a*) Tubercular Enlargement of some of the Lymphatic Glands in the Pectoreal region of the right side. (*b*) The same condition of the glands situated on the inside of the arm near the anterior part of the humero-radial articulation. A l the glands shown in this individual case were well advanced and contained yellow m atter, the larger one having become somewhat caseous.

Before the results of their labors could be communicated to the Pathological Society, Ribbert, of Bonn, published a paper in the *Deutsche Med. Woch.*, 1883, page 413, and announced the parasitic character of the disease and the identity of the bacillus with that described by Koch, from diseased tissues in human tuberculosis. Since this date an admirable article has appeared in the *Journal de L'Anatomie et de la Physiologie*, Tom. XXI, 1885, written by V. Cornil et P. Megnin, entitled "Memoire sur la Tuberculose et la Diphtherie Chez les Gallinaces," containing an account of the microscopical character of the lesions as well as confirming the presence of the bacilli.

Dr. Edwards Crisp has recorded in the Transactions of the Pathological Society, London, several instances of tuberculosis in birds. A few isolated cases have been noted from time to time in the various journals, but no systematic investigation carried out on an extensive scale, as has been attempted, except his own endeavors.

I will here note a brief account from his observations upon zoölogical distributions of the disease. He says that this has received little attention at the hands of Pathologists, and if it were investigated would add a great deal to our stock of knowledge in this direction.

An inquiry into the distribution of tuberculosis among the various classes of the feathered tribe has been of signal service in enabling him to narrow the issue as to the probable origin of the disease.

In his early investigations note was made of the birds which suffered most from the disease, and as has already been mentioned, as far as the farm-yard epidemics were concerned, the grain-eating birds were alone affected. After he was in regular attendance at the Zoölogical Garden, the average monthly mortality was about one hundred, giving a yearly average of 1,200 for five years. From this source alone a good idea of the Zoölogical distribution of the disease could be derived. After one year of work, it became evident that the disease was almost peculiar to graminivorous and fruit-eating birds and vegetable feeders. The affection occasionally occurs in birds of prey, and its presence in them is capable of another explanation.

Birds which live on fish appear to be totally exempt from tuberculosis, and this is also true of water-fowl. Among struthionidæ, the rhea (South American Ostrich) is especially liable to the disease; indeed among thirteen specimens of this bird, which have died since 1881, twelve were thus affected.

Of other species of birds, the common fowl, peacock, guinea fowl, tragopan, grouse, pigeon and partridge are especially liable to tuberculosis. Storks and cranes are not exempt from it.

He further states that opportunities have also occurred for studying the disease in parrots, and some facts of no small degree of importance have been brought to circumstances in the outbreak of the disease in these birds.

Portion of right hind quarter and abdominal wall with skin removed, and (*a*) showing tubercular enlargement of a mammary gland (natural size).

With regard to the cases which have occurred in flesh eating birds, he says, in the early account of avian tuberculosis, published in 1883, the only rapacious birds in which this affection had been detected with certainty were a falcon and an eagle, and that possibly they contracted the disease by feeding on smaller birds affected with tuberculosis. Since that date two other instances have occurred in flesh eaters, an owl and the secretary bird.

The following observation made by J. F. Lachner shows that the disease may occur in wild birds, for he informs us that a sparrow hawk which was taken in a snare presented tubercular modules in the liver, in the lungs and other parts of the body. Goldfinches and other finches are frequently suffererers from this affection, and the hawk may have contracted the tubercules as a result of previous depredation among these pretty birds.

"It is of utmost importance to adduce all the evidence that can be possibly pressed into the services, to strengthen the view that flesh eaters get their tuberculosis by devouring infected grain-feeders, that the following case will be briefly quoted :

In February, 1883, I exhibited the liver of a python, *Python sebæ*, which presented curious nodules in the liver. The details of the case are reported in Vol. XXXIV. of the Path. Society's Transactions, and described as pyæmic abcesses, secondary to a large abcess in the wall of the abdomen, for it was difficult to account for them at that time in any other way that was satisfactory. As soon as bacilli were found in the nodules of the fowls, I at once submitted portions of the liver of the snake, which is preserved in the Museum of the Royal College of Surgeons, to Dr. Heneage Gibbs, to determine whether the bacilli which have been noticed in the lesions of the liver were identical with those in the nodules of the bird's liver.

Examined under improved knowledge derived from a study of the affection in birds, the case came out in a totally new light, for not only were the bacilli unmistakable tubercle bacilli but the microscopical details of the nodules in the snake's liver, harmonized in every way with those characteristics of the avian tuberculosis. Previous to this reëxamination of the specimen by Dr. H. Gibbes and myself, Mr. F. Eve, the Curator of the Pathological Department of the Museum, had rejected the pyæmic view of the lesions and had deposited the specimen in the museum as an example of tuberculosis. This independent testimony is very valuable.

The snake was fed on fowls, pigeons, and ducks, as a rule, and there is, so far as I can see, only one way out of the difficulty. The source of mischief was undoubtedly derived from the alimentary canal ; the birds, pigeons, and fowls are exceptionally liable to tuberculosis, and there can be but little doubt that this was the true source of infection."

## Figures and Tables

**Figure f1:**
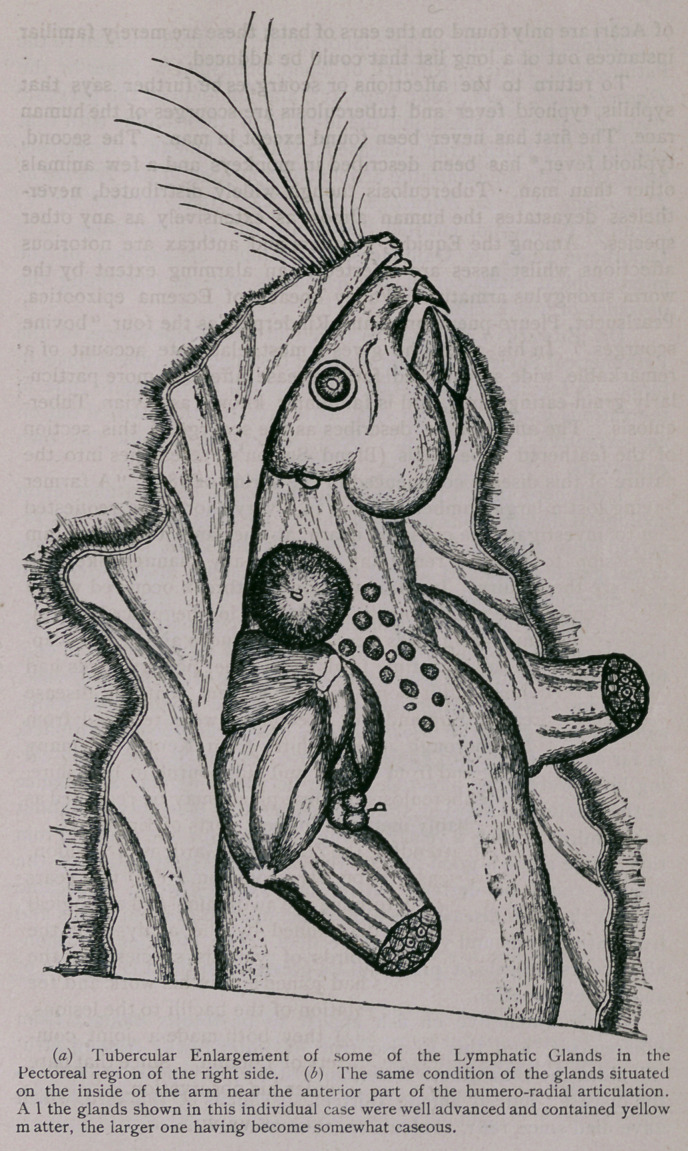


**Figure f2:**